# Neurophysiological Approaches to Lie Detection: A Systematic Review

**DOI:** 10.3390/brainsci15050519

**Published:** 2025-05-18

**Authors:** Bewar Neamat Taha, Muhammet Baykara, Talha Burak Alakuş

**Affiliations:** 1 Department of Software Engineering, Fırat University, Elazığ 23119, Türkiye; bewar_nemat@outlook.com (B.N.T.);; 2 Department of Software Engineering, Kırklareli University, Kırklareli 39100, Türkiye

**Keywords:** EEG, brain computer interface, lie detection, ERP, P300, visual stimuli, recognized face, unrecognized face

## Abstract

**Background and Objectives:** Lie detection is crucial in domains such as security, law enforcement, and clinical assessments. Traditional methods suffer from reliability issues and susceptibility to countermeasures. In recent years, electroencephalography (EEG) and particularly the Event-Related Potential (ERP) P300 component have gained prominence for identifying concealed information. This systematic review aims to evaluate recent studies (2017–2024) on EEG-based lie detection using ERP P300 responses, especially in relation to recognized and unrecognized face stimuli. The goal is to summarize commonly used EEG signal processing techniques, feature extraction methods, and classification algorithms, identifying those that yield the highest accuracy in lie detection tasks. **Methods:** This review followed PRISMA guidelines for systematic reviews. A comprehensive literature search was conducted using IEEE Xplore, Web of Science, Scopus, and Google Scholar, restricted to English-language articles from 2017 to 2024. Studies were included if they focused on EEG-based lie detection, utilized experimental protocols like Concealed Information Test (CIT), Guilty Knowledge Test (GKT), or Deceit Identification Test (DIT), and evaluated classification accuracy using ERP P300 components. **Results:** CIT with ERP P300 was the most frequently employed protocol. The most used preprocessing method was Bandpass Filtering (BPF), and the Discrete Wavelet Transform (DWT) emerged as the preferred feature extraction technique due to its suitability for non-stationary EEG signals. Among classification algorithms, Support Vector Machine (SVM), Linear Discriminant Analysis (LDA), and Convolutional Neural Networks (CNN) were frequently utilized. These findings demonstrate the effectiveness of hybrid and deep learning-based models in enhancing classification performance. **Conclusions:** EEG-based lie detection, particularly using the ERP P300 response to face recognition tasks, shows promising accuracy and robustness compared to traditional polygraph methods. Combining advanced signal processing methods with machine learning and deep learning classifiers significantly improves performance. This review identifies the most effective methodologies and suggests that future research should focus on real-time applications, cross-individual generalization, and reducing system complexity to facilitate broader adoption.

## 1. Introduction

Humans have a long history of using lies to cover up their deceit and wrongdoing. Because of this, discovering ways and applications for detecting falsehoods has been an ongoing subject of inquiry for scientists in this area and a goal for individuals in the last several decades. That is why it is so important to have the means to discern whether someone is being truthful or not. Because of the increasing number of security risks, one of the primary reasons why law enforcement needs lie detectors is so they can determine whether an individual poses a danger to society [1]. Additionally, lie detectors find application in law enforcement, the judicial system, and the fight against crime. Judges use lie-detecting techniques throughout the process of presenting plea bargains that operate in many nations. The community of scientists is currently showing an extensive background in recognizing concealed information, verifying facts and distinguishing falsehoods using a variety of methodologies and strategies utilized to determine falsehood, including counting polygraphs, neuroscience, measurement of peripheral signals that are created by the autonomic nervous system, and EEG signal analysis techniques [1,2].

The polygraph is by far the most frequently used method for detecting unethical behavior because it measures the reaction of the nervous system that controls feelings. Fortunately, its accuracy and dependability vary greatly amongst investigation tasks. Nonetheless, it is quite malleable. Although there has been little advancement in the polygraph’s underlying technology since its development, methods to influence the findings have evolved [2]. Polygraphs quantify cardiac activity and respiratory patterns, so if a person who is taking a polygraph can control their physiological reactions to respiration and cardiac rate, they can influence the test results. It is hard to tell whether a person lies or not during stress, and the outcome of these techniques is neither exact nor admissible in the courtroom. Additionally, in the neuroscience imaging strategy, different viewpoints, such as feeling, conduct, insight, and so forth, as well as cognitive processes and neural activity, are examined. As a result, obtaining the accuracy of two states (untruth and truth) is becoming more difficult [3]. Additionally, functional Magnetic Resonance Imaging (fMRI), which is used half-heartedly, is bulky and necessitates highly specialized skills for the various parts [1,3].

Improvements in the efficiency of analyzing neural data and deciphering brain activity have propelled machine learning (ML) and deep learning (DL) technologies into the rapidly expanding field of computational neuroscience. With the help of a variety of algorithms, ML and DL streamline processing pipelines and enhance learning. For instance, ML algorithms that are supervised learn from training data. Applying the model and learning parameters to fresh or unknown data allows it to anticipate the class labels of the data [4]. Many clinical studies employ binary and multi-label categorizations to examine cognitive function, processing of motor imagery (MI), emotion recognition (ER), and brain diseases such as neurological diseases, brain injuries, and focusing difficulties. When it comes to modelling learning predictions and behavior monitoring, other computational neural models have also been investigated, including the medial Prefrontal Cortex (mPFC) and anterior cingulate cortex (ACC) [5].

**Figure 1:**
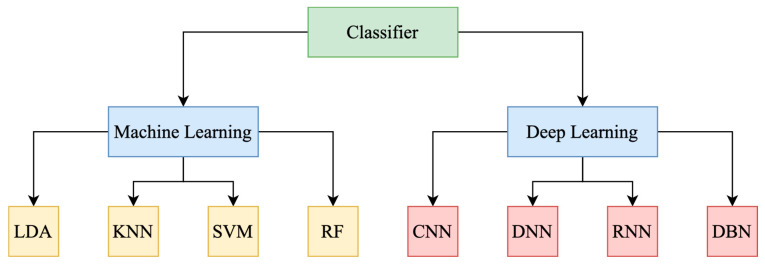
Comparison of ML vs. DL models based on BCI systems.

Nevertheless, lie detection may be made more efficient with the use of DL algorithms. These algorithms are a kind of ML that aims to imitate the human biometric brain. Programs designed to mimic the brain use multi-layered architectures that mimic the communication between neurons in the human nervous system. Layers in DL algorithms can be of many kinds, some examples include logistic and linear regression, activation functions, weights, and classification neural networks. Several ML and DL models have been described to classify EEG signals and identify relevant data, thanks to their remarkable resilience and flexibility [5,6]. There is still a lot of research that needs to be conducted before brain biometrics can be widely used despite their obvious advantages. That is why seven criteria are used to determine the reliability of a biometric as a method of authentication or identification (acceptability, uniqueness, permanence, circumvention, performance, universality, and collectability). In Figure 1, a comparison of AI classification methods between ML and DL algorithms that are commonly used as classifiers in BCI systems, based on the type of input data and various tasks is given [4,5].

About 100 billion neurons and trillions of synapses make up the intricate human brain. After 19th-century scientist Richard Caton began recording rabbit brain impulses, studies of the brain’s electrical activity exploded in popularity. One of the first people to record electrical impulses from the scalp of a human being was Hans Berger, who also recorded brain signals. Traditionally, three main types of brain activity have been identified using EEG signals: brain waves, ERPs and Steady State Visually Evoked Potentials (SSVEPs). Among them, EEG signal processing for various activities most typically uses brain waves [7].

GKT is an additional method that aims to quantify physiological reactions to multiple-choice questions. It can be used to detect concealed knowledge and information. This form of psychophysiological testing is more theoretically solid and morally acceptable than others, including the Control Question Test (CQT). Results from earlier research indicate that a P300-based GKT may effectively leverage the P300 component of ERPs to uncover hidden information [8]. In reaction to important yet uncommon stimuli (oddball stimuli) in EEG brain signals, the P300 component arises. Pattern recognition systems, rather than Bootstrapped Amplitude Difference (BAD) and Bootstrapped Correlation Difference (BCD), are the most suitable instruments for GKT investigations. Since the averaging methodology is utilized to enhance the Signal-to-Noise Ratio (SNR), the BAD and BCD techniques cannot be derived from single-trial ERPs, which is one of their shortcomings. Second, morphological changes in signals may occur while averaging biological signals, which is particularly true in cognitive activities where the latency of signals can alter with time. Consequently, strategies based on single-trial ERPs are appropriate to use. Thirdly, the BAD and BCD do not take phase or frequency information into account since they are based on time domain signal structure. The GKT is different from the traditional polygraph test, which asks direct, accusatory questions [7,8].

**Figure 2:**
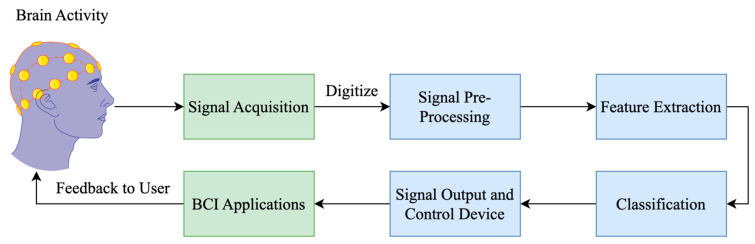
Typical schematic of BCI operation.

Because polygraphs and other methodologies, strategies, and techniques are less reliable for solving this difficulty of lie detection, researchers have investigated various methods for distinguishing between truthful and dishonest speech. The most used method that has been researched for lie detection is using BCI biometric signals [9]. A variety of methods exist for acquiring and producing EEG brain waves. These include implanted methods, evoked potentials (EPs) or ERPs, and operant conditioning, which involve presenting a stimulus to the subject while they are performing a task or activity. The goal of biometrics is to identify people using their unique set of behavioral, physiological, or physical traits, including fingerprints, iris scans, voice, handwriting, and facial features [10]. BCI is a kind of intermodal communication system that converts neural impulses into electrical signals, letting the user operate specialized software or hardware with just the power of thinking. Both clinical settings and daily life may benefit from such an interface for neuromuscular disorders. Acquiring EEG biometric brain signals, processing those signals with preprocessing tools, feature extraction from those signals using classifiers, and identifying publicly available datasets and instruments are the five main components of BCI signals. In Figure 2, a typical schematic of how BCI works is given [9,10].

In addition, EEGs are a noninvasive, low-cost neuroimaging method that may detect false statements about the validity of a statement by capturing the brain’s dynamic patterns at high temporal resolution by means of changes in brainwave patterns [11]. While EEG research has proven useful in many areas, it does have a few drawbacks. For one, the signals are complicated, high-dimensional, nonlinear, and non-stationary. Secondly, the signal-to-noise ratio in the temporal domain is poor, and thirdly, there is inter-individual variability that impacts the performance of analysis and processing [12]. Because of these restrictions, EEG signal processing pipelines are often used. Data preparation to eliminate artefacts, starting the classification method, dataset splitting for the classifier, new data class prediction, and test dataset evaluation are all steps in the standard EEG classification pipeline, particularly in a Magnetic Resonance Imaging (MRI) setting where EEG data are concurrently collected. Consequently, classifying them is a formidable challenge. The subsequent stage involves interpreting the waves, conducting an analysis, and then transforming them into a format comprehensible to humans for integration with other devices [11,12].

EEG is the study of electrical brain activity recorded from a person’s scalp. Electrodes placed on the scalp collect signals from the brain, which are then cleaned using preprocessing techniques such as amplification, sampling, and filtering. After cleaning, their characteristics are retrieved and categorized to ascertain the related kinds of mental operations that the individual carries out. Among the many methods for capturing brain signals, a suitable algorithm uses the identified signals to build a specific application [13]. In the meantime, an EEG is a recording of electrical currents generated in the brain and sent to the scalp. There are five main rhythms in an EEG signal including delta, theta, alpha, beta, and gamma, and they all belong to different frequency ranges, from very low (delta) to very high (gamma). To determine if the person is being truthful or not, EEG equipment is used to capture the neuro signal [14]. There are three main steps involved in collecting EEG: estimating the EEG, recording brain signals, protecting the subject’s information, and analyzing and calculating the results. Based on the EEG designs, two states have been distinguished: misleading and lying. The subjects were responding to the “set of inquiries confined to progress” without regard for the accuracy of their answers. The next step is to capture the subject’s EEG activity while they are carrying out the task. These data will later be analyzed to determine if the subject is telling the truth or lying. Thus, the EEG technique has evolved into a vital resource for many fields at present, and is commonly used in healthcare and diagnostics fields to track and identify various forms of epilepsy, stroke, seizures, sleep problems, brain tumors, traumas, decoding of behavioral activity, and even mortality. The EEG approach, on the other hand, has many more potential uses, such as in fields including entertainment, security, and communication and control [13]. Researchers have been working on several ways to enhance classification and high-quality lie detection using EEG. While there are a few techniques for determining the falsehood of the EEG approach, the problem of lie detection using EEG has emerged in recent years. The reason is that it has the potential to disclose several significant aspects of our thought processes. Recent advances in technology and methodologies have allowed scientists to develop new medical imaging applications, expanding our understanding of how the brain works. Investigating the criminal’s brain might be an additional viable approach, similar to DNA or fingerprints, which have been shown to be effective in identifying them [13,15].

With its clear and undeniable advantage over fMRI in these areas, EEG monitoring of brain activity offers substantial promise as a novel investigative tool in the field of criminal justice, allowing for the linking of connection between a criminal’s memory and the evidence gathered during the crime [1,12]. Therefore, a lie detection system that is based on EEG has the potential to be a more effective instrument, a trustworthy indication of the manner in which thoughts are being organized in the brain, and a technique that is acceptable for detecting a lie, therefore identifying the individuals who are responsible for committing a crime. Due to the fact that they may be used in any circumstance, polygraphs and EEGs offer a huge benefit over more traditional techniques of evaluation. Furthermore, regarding EEG, any effort to trick the system would manifest in the EEG pattern and could be identified. Additionally, it is feasible to examine the asymmetry pattern of resting brainwaves across various levels of intelligence simultaneously to establish the clear activation of the frontal lobe and alpha waves during participants’ state of lying down. The reason for this is that typical lie detection technologies or approaches are not infallible. It has been a few years since this concept was first conceived; however, there is still a great deal of potential for advancement, such as the development of more robust categorization algorithms, improved availability, or reduced prices [12,15].

Due to the lack of a comprehensive review of recent work in this area, this comprehensive study aims to fill that void by surveying recent research efforts on EEG-based lie detection, particularly ERP P300 responses during the presentation of recognized and unrecognized faces, to increase the effectiveness of lie detection for many DL models by examining and comparing EEG data. To build an efficient fraud detection system, lie detector, and truth-finding tool with EEG technology, this review will help researchers compare and analyze present techniques alongside other approaches, find the most common and successful methods, and study them in depth. It will also collect information to understand the available studies, develop, improve, and implement these tools, and make recommendations to make them even more effective. Identifying the gaps that need more research is another key purpose of this study.

The upcoming research review is organized as follows: In the Section 2, we provide and discuss various evaluations of studies related to lie detection. The methodology is detailed in the Section 3, where we explain the approach used to achieve the aims of this study. The Section 4 offers an in-depth discussion of EEG-based lie detection, which is divided into four distinct stages: EEG data analysis, EEG characteristics, effective EEG channels for lie detection, and visual ERP P300. Each of these stages is examined thoroughly. The study’s results are presented in the Section 5, which also includes a discussion that relates the findings to the existing literature. Our research review discussion is further elaborated upon in the Section 6. Finally, the conclusion of our research investigation summarizes the overarching findings.

## 2. Literature Review

This section covers some of the most established methods and algorithms, as many researchers have used various methodologies and conducted numerous tests to detect lies from EEG. Polygraph systems that rely on electroencephalograms include four main parts: data collecting, preprocessing, feature extraction, and final sorting. Several factors affect how well an EEG polygraph system works, including the method of EEG collection, the technology for preprocessing, and the characteristics extracted from EEG signals, and the classification system. What follows is a synopsis of prior research on the topic of hidden information identification for EEG-based lie detection from the reaction into both recognized and unrecognized faces.

In [16], researchers constructed a LieWaves dataset and then analyzed it using methods for DL and signal processing. Using a five-channel (AF3, T7, Pz, T8, and AF4) wearable EEG equipment, they obtained EEG data from 27 participants. The artefacts in the EEG data were removed using the Automated and Adjustable Artefact Removal (ATAR) program. The next step in data augmentation was to use the Overlapping Sliding Window (OSW) approach. In contrast, statistical methods (SM) and the combination of DWT, and Fast Fourier Transform (FFT) were used to examine the EEG signals to extract features. Alongside this, CNN, Long/Short Term Memory (LSTM), and CNN-LSTM hybrid combination algorithm were employed to categorize each feature vector that was acquired. With an accuracy score of 99.88% achieved using the LSTM and DWT approaches, the results showed that the method correctly determined the most accurate result. In a further study [17], the researchers used the ERP P300 to analyze the person’s lying habits and produced a unique scenario for each CIT. The present research used a realistic illegal situation utilizing a 16-channel EasyCap device to collect EEG from 20 volunteers who recognized human faces of both recognized and unrecognized persons. BPF was employed to filter out signal-mixed noise. Researchers used a variety of functional extraction methods across several domains (power, amplitude, complexity, mobility, frequency, and wavelet) to enhance EEG data interpretation. Out of the five classifiers tested, the structure was built by integrating the outputs of the top three using classification evaluation and the Weighted Voting (WV) method: LDA, SVM, and Multilayer Feed Forward Neural Network (MLFFNN). Data classification for guilt or innocence achieved an accuracy of 84.75% using the proposed framework (3-WV) and DWT for extraction. In another study [18], the researchers examined the use of a portable EEG headset to identify lies utilizing ERP data. In the study, researchers examined neurons employed in the recognition of sensitive information as an alternative to the detector test. The test involved 14 channels and 10–20 machines. The first two principal channels contribute to over 80% of data variance and deliver the greatest outcomes. The method used SVM for lie detection is intended to increase system reliability by utilizing fewer EEG signals. An upgraded fraud detection technique constructed around an external EEG recording device and an inexpensive, commonly accessible EMOTIV headset is demonstrated. Frequency parameters retrieved were included, with zeros deleted, including absolute amplitude time window, intensity, and peak-to-peak slope. The technique achieved an accuracy of 83%. In study [19], the researchers suggested a fraud detection method using EEG data from ten participants gathered during CIT for the actual examination of the ERP P300 in recognizing both recognized and unrecognized persons. They employed 16 channels for collecting signals, utilizing different approaches. They introduced a novel cost function in which the Bat method was applied to optimize the SVM parameters, hence improving the classification accuracy of SVM. The Bat binary method was utilized to pick the EEG frequencies. After eliminating ineffective channels in the brain’s temporal lobe, the system’s effectiveness improved to an average accuracy of 96.8%. Researchers in [20], presented falsehoods in the EEG technology and artificial intelligence, investigated the subject’s dishonesty, utilized the ERP P300, and devised a fresh scenario for CIT. ML techniques capable of evaluating EEG data include SVM, K-means, Artificial Neural Network (ANN), and Linear Classifier (LC). The 10–20 method was utilized to record scalp EEG. To analyze EEG data, the categorization model’s properties included signal intensity, wavelength, frequency, and voltage. It used data from the temporal region (T) and the frontal pole (Fp) to identify dishonesty. These parts of the brain are responsible for rational deduction, judgment-related procedures, emotional responses, and mental replay. Simplifying complicated EEG waveforms is the job of the FFT technique, which is used to determine if something is true or false. SVM performed well with a single point of data. Based on a feature for detecting lies, a strategy was 86% accurate. In [21], researchers created an original pattern-recognizing mechanism in responding to the ERP P300 wave that identifies terrible and untainted patients utilizing the GKT method. The goal was to expand the list of attributes with nonlinear components to enhance categorization. Signals were collected from 49 individuals. After performing preliminary processing using BPF, they retrieved features by making use of different frequency bands, wavelet coefficients, and morphological aspects. To choose the best set of parameters, genetic algorithm (GA) was employed. With the LDA classifier and the innovative reactive thresholds approach, the findings indicated that the approach effectively categorized 91.83% of cases by combining fundamental and nonlinear data. In [22], researchers examined the variance in EEG collected through lie detection studies among factual and lying responses from the two categories of respondents utilizing the chaotic phase synchronization (PS) approach. The lie detection test, which followed the traditional three-stimulus technique (TST), was used to collect information from the EEGs of twenty individuals. The lie detection test made use of the phase locking value (PLV) as a statistical measure of PS for specific stimuli. The experiments showed an individual physical and immediate difference in PS due to the liable connecting suffering a stronger/higher PLV compared to the uninvolved group, combined with a high level of accuracy of up to 88.05%. To uncover the true lying mechanism, they examined the widespread frontal-temporal-central-parietal relationship using phase synchronization sequences among 12 EEG data. Regarding this, they researched PS using EEG signals gathered through lie detection research according to some stimuli, as well as PS among EEG activity from various brain areas. Ten undergraduates with no previous experience of mental or neurological illnesses took part in the research. The conductors were configured utilizing the 10–20 method. EEG signals were recorded using 14 methods, including both horizontal and vertical electrooculography (EOG). Researchers in [23], suggested using an ERP P300 EEG signal-based CIT to identify deception in individuals, regardless of whether the faces they see were recognized or unrecognized. Data were obtained from 10 people using a 16-channel EasyCap device. The original EEG data was processed using a BPF. The employed Hjorth criteria to collect data on mobility, activities, and impediments. The K-Nearest Neighbor (KNN) technique was then used to classify these characteristics. Researchers have shown that the median accuracy rate attained utilizing the KNN algorithm was 81.9% after analyzing data collected from specific patients. In [24], authors applied CNN to predict deception. Their main goal was to create an artificial intelligence system that could detect dishonesty using physiological and behavioral indicators. The Dryad dataset was used for training and validation of their proposed model. Six gem photographs served as stimuli for the identification procedure, and thirty individuals were randomly divided into two groups: suspicious and clear. In the suggested model, 12 channels were used for EEG, while the other two were for EOG from AF1 to AF4. In terms of determining whether someone is telling the truth or not, the proposed technique achieved an accuracy of 84.44%. In another study [25], authors proposed the three stages of the CIT classification method that combines Wavelet Transform (WT), K-means clustering, and MLFFNN. To execute the proposed frame, 10 individuals had their EEG data collected utilizing 16-channel EasyCap devices for CIT. The data were then processed using BPF. An accuracy of 83.1% was attained. In [26], researchers explored the classification of EEG data for lie detection by making use of a hybrid combination of traits. A method for obtaining domain properties combined with an SVM classifier is provided in the study. Applying the worldwide 10–20 electrode arrangement technique, the EEG data was gathered at nine electrode places: C3, CZ, C4, P3, Pz, P4, O1, O2, and Oz. Singular inquiry reactions at the sole Pz of the electrode placement were utilized to evaluate the individuals’ exploring actions. A combination of wavelets, duration, frequency, Empirical Mode Decomposition (EMD)-based, and correlating coefficients was employed as characteristics. The EEG data elements that were taken from EMD greatly improved the detection accuracy. Variations in the spatial composition of EEG signals among criminal and free people were revealed through frequency domain features. The dataset has 33 subjects (18 men and 15 women). Three distinct kinds of events were used to collect the data: indifferent (I), targeting (T), and probing (P) events. When the neural data from the 40 various features were put in combination and fed into the SVM, the accuracy for training was 99.94%, the accuracy for testing was 98.8%, and the accuracy for maximum testing was 99.44%. Unconnected reactions from patients to the goal, examination, and additional central electrical deployments might yield helpful data for lie detection. In study of [27], researchers created CIT using the ERP P300 component, and individuals looked at photos of recognized and new faces throughout the trial. Seven out of ten participants’ EEG data were utilized for instruction, while three were tested. The 16-channel EEG cap’s data were preprocessed using BPF, and features were obtained utilizing the Common Spatial Pattern (CSP). Classification performance measures, including LDA, MLFFNN, SVM, KNN, and Naive Bayes (NB) served as the basis for the fuzzy integrator system. Three subjects achieved an average classification accuracy of 100%. For data acquisition in the frequency and temporal domains, the researchers also used a DL method based on a restricted Boltzmann machine with a wavelet. They used ERP P300 wave analysis using EEG data obtained during CIT using a 16-channel EasyCap device by showing participants pictures of both familiar and unfamiliar faces. The EEG data were preprocessed using BPF so that they could be utilized for WT analysis. A median accuracy of 81.03% was achieved when a Deep Belief Network (DBN) was used to determine whether the brain waves of 10 subjects were genuine or artificial. In study [28], authors offered research participants the electroencephalogram-based DIT. Face recognition ability with familiar and unidentified faces was tested on the ERP P300. EEGs of 20 participants were recorded for the trial. After BPF processing, 16-channel EasyCap signals were wave-pegged. Researchers used characteristics into LDA classification using Wavelet Packet Transform (WPT)-derived equations. The WPT-LDA approach for dishonesty detection obtained a mean classification accuracy of 91.67%. In [8], researchers used BPF as a preprocessing technique from a sample of 20 individuals prior to analyzing the ERP P300 before developing CIT. They used a 16-channel EasyCap device to gather data on the participants. In the study, participants’ emotions were recorded in regard to images of famous people and those they knew. This was followed by the extraction of EEG signal properties using the Short-Time Fourier Transform (STFT) technique. The researchers used binary Bat to choose the most appropriate collection of routines. To train the Extreme Learning Machine (ELM) to distinguish between guilty and innocent individuals, the gathered collection of traits was later input into it. The proposed lie-detecting system was a success, with 88.3% accuracy. 

Table 1 provides an overview and comparison of various hidden information identification approaches for lie detection using EEG in the context of ERP P300 in response to recognized and unrecognized faces.

## 3. Methodology

**Figure 3:**
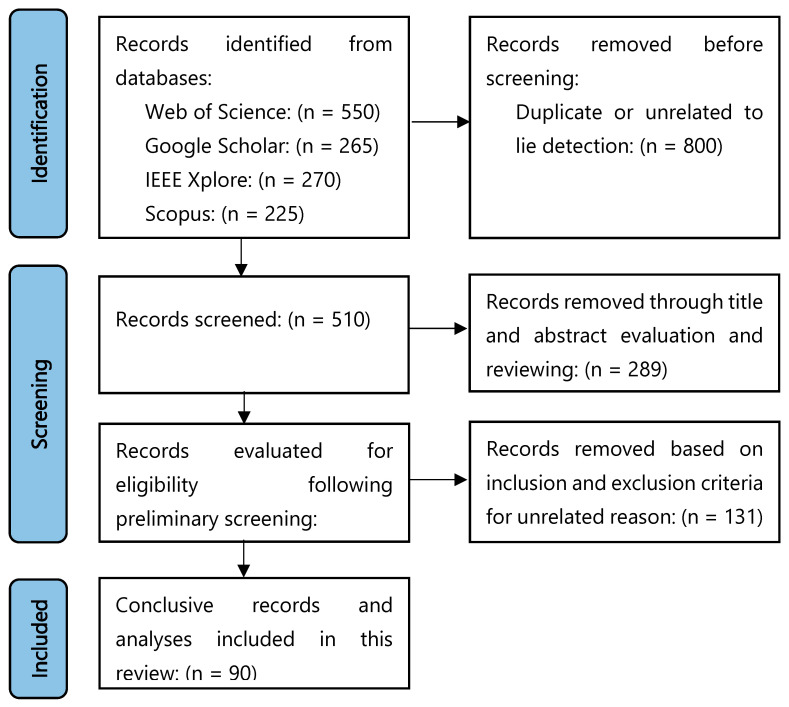
PRISMA review structure implemented for this review.

This review adhered to the Preferred Reporting Items for Systematic Reviews and Meta-Analyses (PRISMA) guidelines to maintain methodological reliability, reproducibility, reliability and rigor. A comprehensive search was performed different electronic databases including IEEE Xplore, Web of Science, Google Scholar and Scopus. The search was restricted to English-language journal articles and conference papers. Relevant studies were located using the keyword string: [(Lie) AND (EEG OR electroencephalography OR detection) AND (brain signal) AND (classification OR detection)]. To broaden the scope of the search, synonyms such as “deception” and “face recognition” were also included in the connectivity analysis. This search was designed to identify research focused on lie detection, particularly through studies related to the classification, prediction and detection of EEG signals. The search was conducted within the title, abstract and keyword sections. A graphical abstract of the entire search and screening process is presented in Figure 3.

The methodology employed to achieve the research aim is explained in this segment. The main goal of this study was to provide a thorough review of the current procedures and techniques used for extracting and classifying data, together with the resultant levels of accuracy, for specific research published between January 2017 and February 2024. The search terms used to identify relevant articles between these dates varied depending on the datasets scanned.

The search queries for IEEE Xplore are as follows: ((“EEG” AND “lie detection”) AND (Publication Year: 2017 TO 2024) AND (Content Type: Journals OR Conferences) AND (Language: English)). For Scopus: (TITLE-ABS-KEY(“EEG” AND “lie detection”)) AND PUBYEAR > 2016 AND PUBYEAR < 2025 AND (LIMIT-TO(DOCTYPE, “ar”) OR LIMIT-TO(DOCTYPE, “cp”)) AND (LIMIT-TO(LANGUAGE, “English”)). For Google Scholar: Search: “EEG” AND “lie detection”, Custom range: 2017–2024. Finally for Web of Science: TS = (“EEG” AND “lie detection”) AND PY = (2017–2024) AND LA = (English) AND DOCUMENT TYPES: (Article OR Proceedings Paper). Nonetheless, we concentrated on and compiled a comprehensive summary of the most pertinent information sources in this area, which was assembled to accomplish the primary goals of the review and survey. Additionally, for finding the best source and articles to achieve the main objective results and preparing this survey from the mentioned source, we used those queries for our research results, such as EEG signal-based lie detection, the DL method used with the EEG signal to identify lie detection, and the best way for lie detection with the EEG signal by using DL methods. During the last seven years, a selection of the most relevant studies analyzing the ERP P300 component’s response to human faces for EEG-based lie detection has been made.

### Study Inclusion and Exclusion Criteria

After the article search was completed, titles and abstracts of all retrieved records were manually reviewed by the authors. Full-text articles were assessed for eligibility according to predefined inclusion criteria as listed below:

- English journal articles and conference proceedings analyzing lie detection based on EEG signals were included as the inclusion criteria.
- The selection was made according to the feature extraction methods based on signal processing studies.
- Articles focusing on the accuracy metrics of the models’ performance were taken into consideration.
- Studies analyzing brain responses to recognized and unrecognized faces, particularly involving the ERP P300 component, were included.
- Studies that included experimental protocols (e.g., CIT, GKT, DIT) in which EEG signals obtained from humans were included.

For the purposes of this review, the following article topics were excluded from consideration:

- Studies using different approaches (e.g., fMRI, polygraph, peripheral bio signals) other than EEG were not evaluated.
- Studies that were not directly related to lie detection (e.g., only measuring attention, stress, depression) even if EEG was used, were excluded.
- Theoretical studies that only described EEG collection methods, electrode placement, or general EEG signal properties were excluded.
- Research that did not report or compare metrics such as classification accuracy, F1 score, etc. was excluded.
- Studies, whose full text could not be accessed, even if the title and abstract were appropriate, were excluded.

## 4. EEG-Based Lie Detection

### 4.1. EEG Data Analysis

The interpretation of scalp-recorded electrical activity of the brain requires a sophisticated procedure known as EEG signal data analysis. Every individual component is critical for the effective processing of data and must be resolved in that order. This analysis encompasses a sequence of procedures, commencing with data acquisition and concluding with interpretation. Its applicability extends to numerous disciplines, such as medicine, psychology, neuroscience, and diagnostics. The section below is an exhaustive synopsis of the data analysis procedure [30].

#### 4.1.1. Data Acquisition

EEG terminology denotes the method by which the electrical activities produced by the brain are captured and documented. This involves several key steps and components: electrode placement and EEG electrodes are systematically positioned on the cranium in accordance with standardized systems such as the International 10–20 system. Their purpose is to sense the electrical activity produced by neurons within the brain. Furthermore, during Analog to Digital Conversion (ADC), the continuous EEG signal is sampled at a high specific rate (sampling rate or frequency). Common sampling rates range from 256 Hz to 1024 Hz to capture the rapid fluctuations in brain activity, which means that the signal is measured at 256 to 1024 times per second. In addition to amplification, the captured electrical signals are very weak and require amplification; EEG amplifiers boost the signal strength to a level that can be effectively processed and analyzed [30].

Moreover, in signal detection, the electrodes capture the electrical signals, which are typically in the range of microvolts (μV); these signals represent the spontaneous electrical activity of the brain. Furthermore, ADC involves the utilization of an analogue-to-digital converter to transform filtered and amplified analogue signals into digital format. This conversion enables the signals to be subjected to digital computing techniques for analysis and processing [30,31].

In addition to data storage, the digitized EEG signals are stored in a data storage system for further analysis, and the data can be stored locally on the EEG devices or transmitted to a remote storage system [31]. Moreover, data processing and analysis to get useful information from stored EEG data are processed and analyzed in several ways. These involve signal preprocessing like noise reduction, feature extraction (finding relevant patterns or traits), and more developed analytical techniques involving time-frequency analysis or artificial intelligence methods. Finally, in terms of visualization, some software programs can turn processed EEG data into pictorial images of the brain’s electrical activity. These can be seen as waves or topographic maps [32].

#### 4.1.2. Preprocessing

In EEG, waves are processed through a set of steps that prepare the raw EEG data for further study [33]. This step is very important to improve the data’s quality, eliminate noise and other errors, and make the data usable for correct reading and further computer studies [34]. Important steps in EEG data preprocessing include filtering, which involves applying various filters to remove unwanted noise, artefacts, and frequency components from the EEG signal, including a high-pass filter that eliminates low-frequency noise such as drift, a low-pass filter to remove high-frequency noise such as muscle artefacts activity, eye movements or external electrical interference, and a band-pass filter to retain only frequencies within a specific range [30,35].

However, artefact removal is unwanted signals that can contaminate EEG data. Common sources of artefacts include eye blinks, EOG, muscle activity, and electrical interference. However, there are some techniques that can remove artefacts, including ICA, in which the EEG signal is broken up into statistically different parts that can be used to find and eliminate errors; regression methods that can subtract artifact-related activity from the EEG signal; and manual inspection and removal; as well as visual identification and exclusion of segments with artefacts [32,36]. Furthermore, re-referencing EEG signal steps are often recorded with respect to a reference electrode, with the purpose of enhancing signal quality while decreasing noise, while one must alter the reference. The common re-referencing methods include the Common Average Reference (CAR) to average the signals from all electrodes and use this average as the reference. Linked mastoids or ears use the average of two electrodes placed on the mastoids or ears as a reference [37].

In addition, segmentation (epoching), involves dividing the continuous EEG data into smaller segments or epochs. This step is often synchronized with specific events or stimuli and is essential for ERP analysis. Baseline correction, prior to analyzing epochs, is important to correct for any baseline shifts, and is performed by subtracting the average signal during a pre-stimulus period from the entire epoch [38]. Additionally, down sampling, which lowers the EEG data sample rate to lessen the computing burden while keeping the crucial signal properties, is often necessary when the initial sampling rate is very high. Finally, the normalization step to standardizing the EEG data to a common scale to facilitate comparison between different datasets or subjects can involve Z-score normalization or other statistical methods [38,39].

#### 4.1.3. Feature Extraction

The phrase “signal processing” is utilized to describe the steps used in EEG to extract useful information from the raw EEG data to perform tasks like diagnosis, classification, or BCI. This process is crucial because most raw EEG data are complicated, noisy, and multidimensional, making direct analysis and interpretation challenging. Feature extraction facilitates data reduction by keeping key information intact while reducing the data’s dimensionality [40]. At the same time, there are six basic concepts involved in feature extraction for EEG signals: the time domain, the frequency domain, the time-frequency domain, the spatial domain, the non-linear, and the connection features. However, each of these concepts contains some other important features [41].

The time-domain features contain statistical parameters of the EEG signal, like mean, variance, standard deviation, skewness, and kurtosis, thereby providing knowledge about the pattern of distribution and shape of the signal. In addition, amplitude features involve extracting the maximum, minimum, and Peak-to-peak (P2P) amplitudes of the EEG signal. Finally, the zero-crossing rate indicates the frequency content of the EEG signal and is measured as the rate at which the signal passes the zero voltage thresholds. While the frequency-domain features contain the power spectral density (PSD), which depicts the power distribution of the EEG data across various frequency bands; the most popular way to calculate PSD are the FFT and Welch’s technique [41,42]. Furthermore, DWT and FFT are two mathematical methodologies for signal analysis. They vary in computing expense and susceptibility to noise. The FFT exhibits a reduced computational complexity, often *O(NlogN*), where *N* represents the quantity of data points. The FFT is designed to effectively calculate the DFT using data symmetries, enhancing speed for extensive datasets. The DWT incurs a computing cost of *O(N)* for single-level decomposition, with *N* being the number of data points. Nonetheless, it is often more computationally demanding than the FFT for extensive datasets because of the multiple-scale operations required, particularly at elevated degrees of decomposition. Nevertheless, the FFT exhibits reduced sensitivity to noise for the preservation of high-frequency components. It operates well when the signal is mostly composed of periodic elements. Nonetheless, it may encounter difficulties with noise since it disperses the signal’s energy over the frequency range, involving noisy frequencies. DWT is often more efficient in managing noisy signals due to its provision of time-frequency localization. Analyzing the signal over several scales enhances the ability to distinguish significant characteristics from noise, particularly in non-stationary or localized noise. However, there is band power, which refers to the strength within certain frequency bands, such as delta, theta, alpha, beta, and gamma, linked to distinct mental states and cognitive operations. Lastly, we have spectrum entropy, a metric for the EEG signal’s spectrum difficulty [42,43].

The time-frequency domain features incorporate the WT, which provides both time and frequency information by breaking down the EEG signal into various frequency components with variable precision. Moreover, the STFT offers the temporal development of the signal’s frequency content [44]. In contrast, channel selection is a part of Spatial Domain Features (SDF) that helps choose useful EEG channels according to how they contribute to the job at hand. Moreover, CSP is a method for improving EEG signal discrimination by locating spatial filters that increase the variance disparity across two signal classes [37,45]. Furthermore, non-linear features include fractal dimension (FD) to measure the complexity of the EEG signal. Moreover, the Lyapunov exponent indicates the predictability of the EEG signal by measuring the distance between two very close trajectories. Finally, connectivity features (CF) include coherence to measure the degree of synchronization between EEG signals from different channels [46,47].

In contrast, feature extraction is important for dimensionality reductions. By focusing on the most informative features, it reduces the amount of data to process, making computations more efficient. In addition, noise reduction helps to eliminate irrelevant and noisy components of the EEG signal [48]. Moreover, improved classification and interpretation enhance the performance of machine learning algorithms in tasks like emotion recognition, seizure detection, and BCI by providing more discriminative features. Finally, it provides information on brain activity because the extracted features can provide insights into different cognitive states and neurological conditions [49,50]. However, we can significantly overview and compare several algorithms by computational complexity, average time complexity, worst-case complexity, noise robustness, and real-time applicability, as shown in Table 2 below [51,52].

#### 4.1.4. Pattern Recognition and Classification

Pattern recognition refers to the automated identification of regularities and patterns within EEG signals. Several phases are involved in this process, beginning with signal capture and continuing through preprocessing, feature extraction, and pattern recognition, which utilizes algorithms to detect patterns within the extracted features [53]. Those techniques can include several methods such as SM, ML and DL models. At the same time, classification in EEG signals involves categorizing the identified patterns into predefined classes or categories. This process can be described as follows: the selection of classifiers, the choice of appropriate classification algorithms based on the nature of the EEG data, and the classification task [54]. Nonetheless, LR and LDA are two examples of LC that are often utilized for selection criteria. Applications of non-linear classification methods include KNN and SVM. Collective techniques include boosting algorithms and RF [55]. Lastly, there are neural networks, which include models like Recurrent Neural Network (RNN), CNNs, and other DL architectures [56].

However, other classification categories involve training that uses labelled EEG data where the correct classification is known to train the classifier, which consists of adjusting the algorithm’s parameters to minimize classification errors. There is also testing and validation, which involves assessing the classifier’s accuracy on new data to make sure it can generalize correctly and not become too reliant on the training set. Common approaches for this include cross-validation. Finally, the application applies the trained classifier to new, unlabeled EEG data to predict the class or category. This can be used in different applications, such as the identification of epileptic convulsions and medical diagnoses, sleep disorders, or other neurological conditions. BCIs are the ability to communicate and exercise agency for people with impairments [57,58].

Finally, cognitive and emotional state monitoring is used to assess mental states such as attention, relaxation, or stress. However, the combination of pattern recognition and classification in EEG signals has significant implications across various fields, including healthcare, to enhance diagnostic accuracy for neurological and psychiatric conditions [59]. Neurofeedback provides real-time feedback for cognitive and emotional self-regulation. Human–computer interaction (HCI) improves the functionality and user experience of BCIs. Also, research advances our understanding of brain function and dysfunction [60,61].

#### 4.1.5. Connectivity Analysis

In EEG, signals refer to the study and measurement of the functional or effective connections between various brain areas as reflected in EEG recordings. Analyses of this kind seek to deduce the mechanisms by which different brain regions interact and work in tandem. There are several key concepts and methods involved in the connectivity analysis of EEG signals.

Functional connectivity calculates the statistical dependence of EEG data from various brain areas. Correlation analysis, which looks at the linear connection among EEG signals, is one of the popular approaches to functional connectivity. Coherence evaluates how consistently the phase variance of EEG signals varies with frequency. The consistency of phase discrepancies between signals is measured by the PLV. Finally, mutual information evaluates the dependency between signals without assuming a linear relationship [62]. Additionally, another key concept of connectivity analysis is effective connectivity, which emphasizes how one brain system affects another causally. However, the common methods of effective connectivity include Granger causality, which investigates the possibility of predicting future values of one time series from its previous values. Dynamic Causal Modeling (DCM) uses a mathematical model to infer and quantify directed interactions among brain regions. Finally, transfer entropy that measures the directed information transfer between signals [63,64].

While the main applications involved in the connectivity analysis of EEG signals include clinical diagnosis and monitoring, which identify abnormalities in brain connectivity that may be associated with nervous system conditions like epilepsy, schizophrenia, autism, and Alzheimer’s disease, cognitive neuroscience involves understanding brain mechanisms underlying cognitive processes such as attention, memory, and perception. Finally, BCIs develop systems that interpret brain signals to control external devices. Moreover, the challenges faced in the connectivity analysis step of EEG signals include the volume conduction effect, which is the challenge of distinguishing true brain connectivity from spurious correlations due to volume conduction; high dimensionality manages the complex, high-dimensional nature of EEG data. Finally, inter-subject variability accounts for differences in brain anatomy and function across individuals [65,66]. Furthermore, discoveries on functional connectivity have demonstrated considerable potential in deception detection, especially by examining brain activity patterns during deceit. Functional connectivity denotes the visual connection of geographically remote brain areas, typically assessed by neuroimaging methodologies such as fMRI or EEG. These findings are significant as they indicate that brain networks exhibit increased or decreased activity during deceitful conduct. Key results from functional connectivity research that might improve practical deception detection models include heightened prefrontal cortex activity, as deceit frequently necessitates a greater cognitive burden, including the suppression of true replies or the fabrication of facts. This results in heightened activity in regions such as the prefrontal cortex, which is associated with executive functioning, working memory, and decision-making. Secondly, research indicates that deceptive conduct modifies the connection among brain areas related to cognitive control, emotional regulation, and memory retrieval. The prefrontal cortex might show an increased connection with areas like the ACC which plays a role in mistake detection and emotional control. Thirdly, stress responses and emotional regulation can be elicited by deceptive activities, potentially resulting in physiological and emotional reactions, such as elevated heart rate or anxiety, which may influence brain connections, particularly in areas such as the amygdala and insula. These areas participate in emotional processing and stress reactions, and their connection patterns may provide further insights for deception detection models. Ultimately, temporal dynamics, including activation timing and connection patterns across time, may be crucial for differentiating between true and false responses. For instance, deceitful individuals may have modified temporal dynamics, including delayed or less coordinated activity within various brain networks [63,64,65,66].

#### 4.1.6. Interpretation and Applications

The interpretation of EEG signals involves deciphering the timbres and patterns of these electrical impulses to understand brain function and diagnose neurological conditions. However, the key elements in interpreting EEG signals include waveforms and frequencies because EEG signals are characterized by various waveforms, each associated with different states of brain activity [67]. Another key element is spatial distribution because the location of electrical activity on the scalp can provide insights into which parts of the brain are active or affected, among which is the prefrontal cortex, which oversees executive functions like emotion regulation, decision-making, and problem-solving. The temporal lobe is associated with memory and auditory processing. The parietal lobe is elated to sensory information and spatial awareness. Ultimately, the processing of visual information is handled by the occipital lobe [68,69].

**Figure 4:**
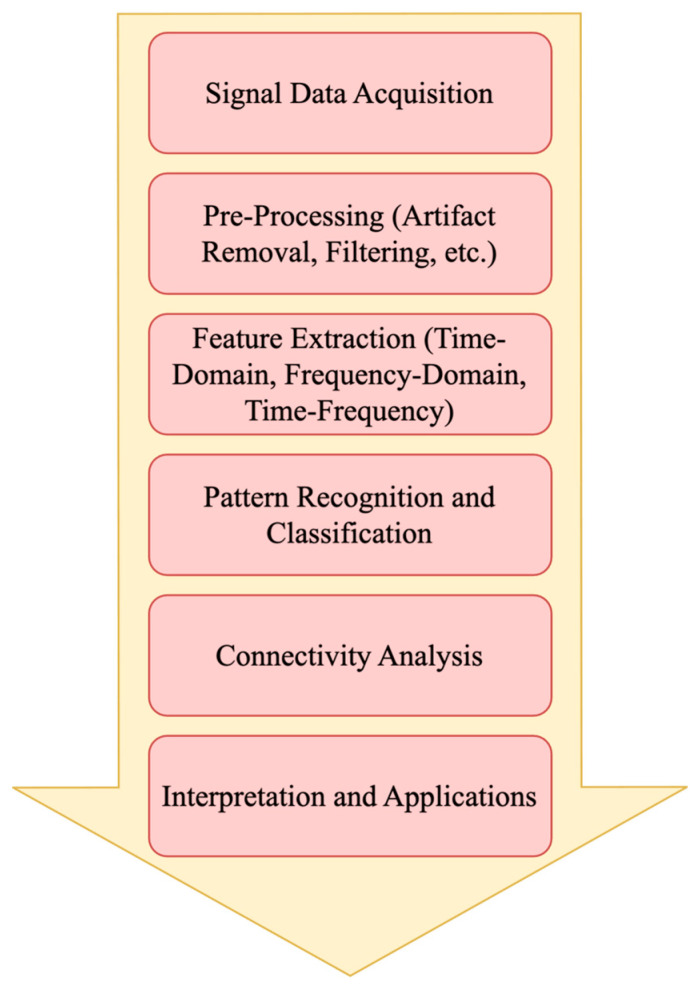
A high-level illustration of how EEG data are analyzed.

Moreover, other key elements are patterns and abnormalities because the specific patterns can indicate normal or abnormal brain function, which include epileptiform activity such as spikes, sharp waves, and complex waveforms that may indicate epilepsy. In addition, asymmetries, which are differences in electrical activity between the two hemispheres, can suggest localized brain lesions or focal disorders [69]. However, numerous clinical and scientific contexts and applications make use of EEG signals. The clinical diagnosis applications include epilepsy because EEG is crucial in diagnosing and localizing epileptic foci. Sleep disorders are used in sleep studies to diagnose conditions like sleep apnea, insomnia, and narcolepsy. Brain injuries help assess the extent of brain damage after traumatic injuries or strokes. Finally, neurodegenerative diseases can aid in the diagnosis of conditions like Alzheimer’s disease by detecting abnormal patterns [70]. Additionally, cognitive and behavioral research applications include cognitive function because studying EEG patterns can provide insights into processes like attention, memory, and learning, as well as emotion and mood, because EEG can help understand the neural correlates of emotions and mood disorders [70]. Furthermore, the neurofeedback and BCI applications include neurofeedback, where people may train themselves to regulate their brain waves, which can be beneficial for conditions like Attention Deficit and Hyperactivity Disorder (ADHD), anxiety, and depression [71]. Also, BCI allows the brain and other gadgets to communicate directly with one another, which can assist individuals with disabilities in controlling prosthetics or communication devices. In contrast, monitoring and prognosis applications include an aesthesia monitoring because, during surgery, EEG is utilized to monitor anesthetic depth. Also, coma and vegetative states provide prognostic information about recovery potential in patients with severe brain injury [72]. Additionally, neuromarketing and consumer research and preferences in EEG are used to understand consumer responses to products and advertisements by analyzing their neural responses. Figure 4 represents a high-level illustration of the analysis process for EEG data [68,71,73].

### 4.2. EEG Characteristics

**Figure 5:**
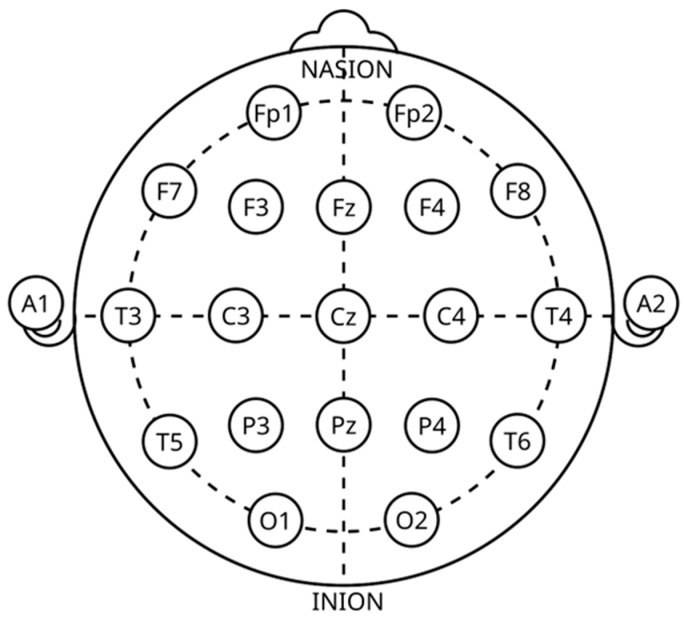
Positioning of electrodes based on the 10–20 international system.

According to the 10–20 worldwide guidelines, as indicated in Figure 5, EEGs capture data by placing electrodes on the head, directly over the brain. Five distinct frequency bands make up an EEG waveform: alpha, beta, theta, delta, and gamma. Each of these bands is briefly detailed here [1,73,74,75]:

i. **Delta (0.5–4 Hz):** When people are deeply and unconsciously asleep, the slowest EEG waves are usually seen. At this point, delta waves have sizable amplitudes (75–200 volts (V)) and are thus thought to represent the person’s unconscious mind. As our sensitivity to the external environment declines, delta waves become more pronounced [75].
ii. **Theta (4–8 Hz):** Experiencing waves is best produced when one is calm and focused. Additionally, one may see them while relaxing, in light sleep, recalling memories, and even on certain tests for short-term memory. They can also appear in some pathological circumstances. An amplitude of 100V is typical for theta waves [1,75].
iii. **Alpha (8–14 Hz):** The most typical patterns in individuals who are physically and mentally well. While the person is awake and relaxed, alpha waves have been identified on rare occasions with closed eyelids [1,75].
iv. **Beta (14–30 Hz):** Beta waves are linked to heightened awareness, proactive cognition, anxious contemplation, and focused concentration. 10V amplitude characterizes these waves [1,73].
v. **Gamma (Above 30Hz):** These are formed during extended high-level information processing. With amplitudes of less than 2V, these waves are very tiny. When it comes to visual inputs, cognitive functioning, and image understanding, gamma waves are recognized to have more important electrical impulses [73,75].

### 4.3. Effective EEG Channels for Lie Detection

Research has shown that specific EEG channels are particularly effective in identifying deception-related activity, especially in the frontal cortex, prefrontal cortex (PFC), frontal poles, midline frontal region, parietal cortex, temporal and central regions, occipital regions, and ACC. These channels are more efficient at distinguishing between truthful and deceptive responses and correspond to specific scalp locations based on the 10–20 electrode placement system [1,73,74,75]. Moreover, the frontal cortex plays a significant role in working memory, higher-order thinking, cognitive load detection, and conflict processing. Key areas within the frontal cortex include F3, F4, Fz, FCz, FC3, and FC4. Additionally, the PFC is vital for decision-making, cognitive control, and executive functions. Engaging in deception often requires more mental effort, activating the frontal regions of the brain. This activation occurs because lying necessitates response inhibition and the manipulation of information. Certain areas are particularly relevant in this context. The frontal poles (Fp1, Fp2) are linked to emotional regulation and executive functions during deceptive acts. Meanwhile, the midline frontal region (Fz) is involved in attention and error monitoring when fabricating lies [74,75]. Furthermore, the parietal cortex contributes to attention and sensory integration, processing memory-related deceptions. This region exhibits distinct wave responses associated with the recognition of concealed information, including areas P3, P4, and Pz. Moreover, the temporal and central regions monitor motor-related responses, and the temporal areas handle language processing and memory retrieval, both of which are essential for constructing deceptive replies. These include locations Cz, T3, T4, T5, and T6 [1,75].

**Figure 6:**
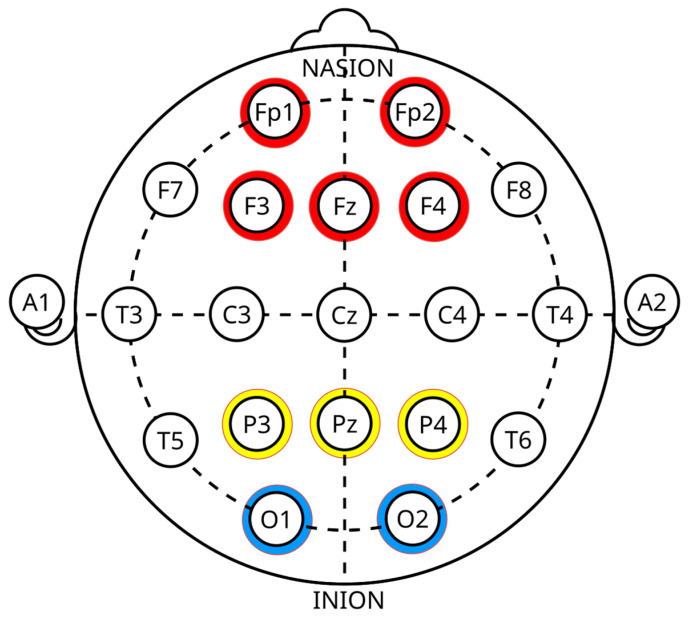
Head map of effective lie-detecting EEG channels.

Although less frequently used but sometimes examined when visual stimuli are involved in the deception process, the occipital regions key areas include O1 and O2. In addition, the ACC crucial for conflict monitoring and error detection, showing high activity during deceptive situations. Key locations within the ACC include FCz, Fz, and Cz. Notably, the frontal-central region is critical for detecting conflict related to deception and for response inhibition [1,73]. However, the EEG head map highlights the most effective channels for lie detection. The regions with high activity during deception are marked in red with frontal and prefrontal channels, including Fp1, Fp2, F3, F4, and Fz. Moderate activity is marked in yellow with parietal channels, including P3, P4, and Pz. Baseline regions are marked in blue with occipital channels, including O1 and O2 [1,73,74,75], as indicate in below Figure 6.

### 4.4. Visual ERP P300

Brain cells (neurons) that are in communication with one another produce minuscule electrical signals that are detected by electrodes inserted in the cranium. An assortment of brain potentials is produced in response to various stimuli. One of them ERPs; specific patterns and significant components used from EEG signals to measure the brain activity that is time-locked to a particular event or stimulus [18]. Furthermore, it is an automatic psychological response that occurs in the human brain because of a reflex, which the analysis of EEG data may quantify during the processing of motor, sensory, or cognitive information. ERP has been used to identify brain activity patterns linked to fraudulent details, making it the main and most often employed technique for discovering hidden information [26]. The P300 wave component is a well-researched event-related potential that is characterized by a positive component in the ERP. The P300 response is characterized by a positive waveform deviation in the EEG signal that occurs about 300 to 1000 milliseconds after the stimulus is presented. The brain’s reaction to uncommon and important inputs is distinct from its response to irrelevant stimuli and is related to activities like attention, recognition, and working memory. Assessing the magnitude of the P300 wave is used to evaluate if the person is concealing information [18,26].

The most important characteristic of the P300 wave includes latency because around 300 milliseconds later, the P300 peak displays itself after the presentation of the target stimulus, and the exact timing can vary depending on the task and individual differences. Amplitude results because the amplitude of the P300 wave varies based on factors like the relevance or salience of the stimulus, the subject’s attention, and cognitive capacity. Finally, scalp distribution, when the P300 is usually largest over the parietal and central regions of the scalp can be observed across various electrode sites. The P300 is part of the ERP and is typically elicited in decision-making processes where the subject needs to detect a rare or significant event within a series of stimuli [2,76]. Whatever it is about types of stimuli from a visual ERP P300 study, the stimuli are usually visual, such as images or flashing lights. The subject might be asked to respond to an infrequent target stimulus amid frequent non-target stimuli such as the “oddball paradigm”. It is the most common experimental design to elicit a P300. The individual is exposed to a series of visual stimuli, wherein infrequent target stimuli, such as a specific image, are interspersed with frequent non-target stimuli, such as different images. Then, the subject is instructed to respond, such as by pressing a button when the target stimulus appears [77,78].

On the other hand, the P300 is linked to cognitive operation-like focus, working memory, and expectancy updating, and is thought to reflect the allocation of attentional resources and the updating of the cognitive context [79]. In addition, abnormalities in P300 are used in the diagnosis and monitoring of conditions such as schizophrenia, ADHD, and dementia. Furthermore, P300 is utilized to study cognitive development, aging, and various aspects of mental functioning. Finally, the P300 is used in BCI systems to facilitate movement and speech for those who have profound motor disabilities; by detecting the P300 response to specific stimuli, a computer can interpret the user’s intentions [80]. Due to the above important reasons, the visual ERP P300 is a powerful instrument for detecting deceit since it stands out for its peak performance in uncommon occurrences and provides the opportunity for trustworthy, countermeasure-proof lie detection. Due to the P300 component’s sensitivity for covert face recognition, prior research has shown the faces that may be utilized as stimuli within the context of the ERP P300 in establishing an efficient lie detection system. Lie detection based on P300 may be more effective when presented with recognized or unrecognized visual stimuli [81,82].

## 5. Results

**Figure 7:**
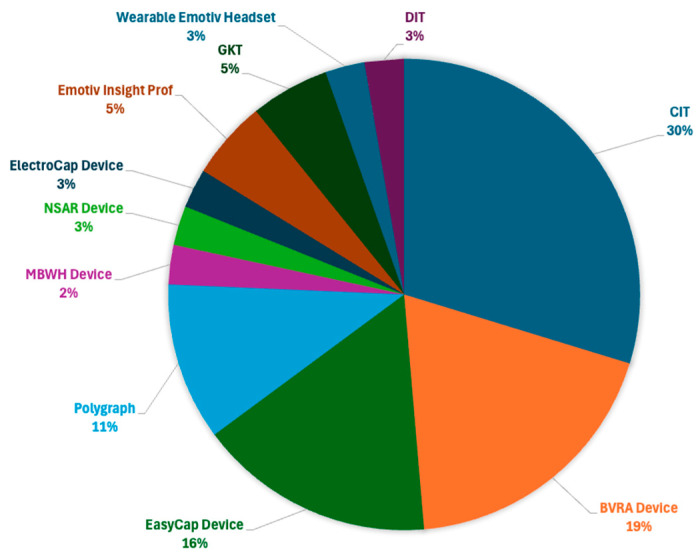
The most used protocols from 2017 to 2024.

This study indicates that the CIT technique is the most often used approach for assessing an individual’s behavior while lying. This method utilizes the ERP P300 paradigm, which measures brain activity in reaction to visual stimuli of recognized and unrecognized faces using EEG data, as indicated in Figure 7.

**Figure 8:**
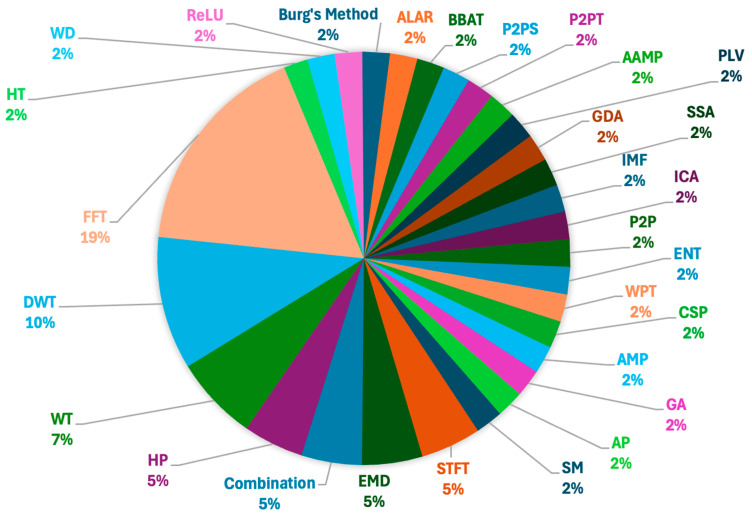
The most often-applied feature extraction techniques ranged from 2017 to 2024.

In addition, the FFT and DWT approaches were the most often used techniques for extracting features in EEG-based lie detection for hidden information identification, as indicated in Figure 8. However, DWT stands out due to its exceptional ability to process non-stationary signals, such as EEG data. It offers multi-resolution analysis, which allows for effective segmentation into time-frequency components. This capability makes DWT suitable for capturing transient patterns in EEG data, such as epileptic spikes or different sleep phases. In contrast, alternatives like EMD are less commonly used. This is primarily because they tend to be computationally intensive, less stable, and highly dependent on the data. Furthermore, EMD often encounters issues such as mode mixing and lacks a solid theoretical foundation. For real-time or large-scale EEG analysis, where robustness and efficiency are crucial, EMD proves to be less viable due to these limitations [16,17,18,19].

In addition, the BPF method has been mostly used for preprocessing purposes, and in 87% of the studies examined, it was observed that the signal quality was preserved by allowing only a certain frequency range and fading values outside this range. 

**Figure 9:**
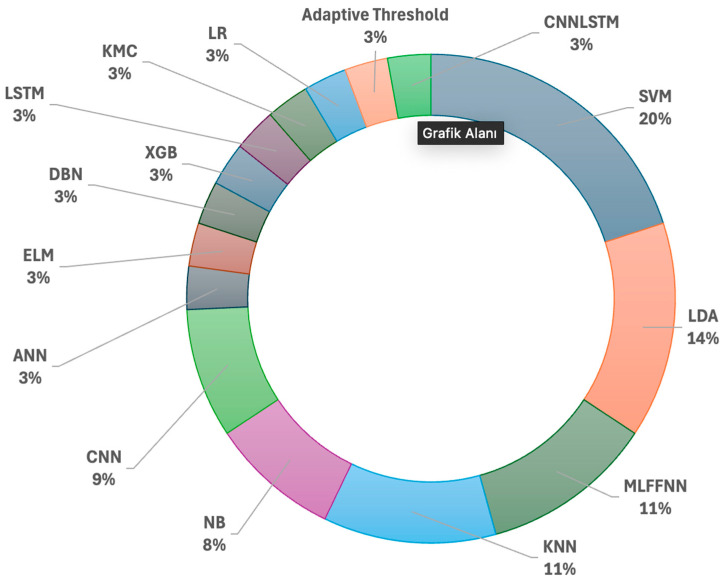
The most often-used classification techniques ranged from 2017 to 2024.

For the classification purposes, it is seen that in Figure 9, the most often classifiers are SVM, LDA, MLFFNN, and KNN.

**Figure 10:**
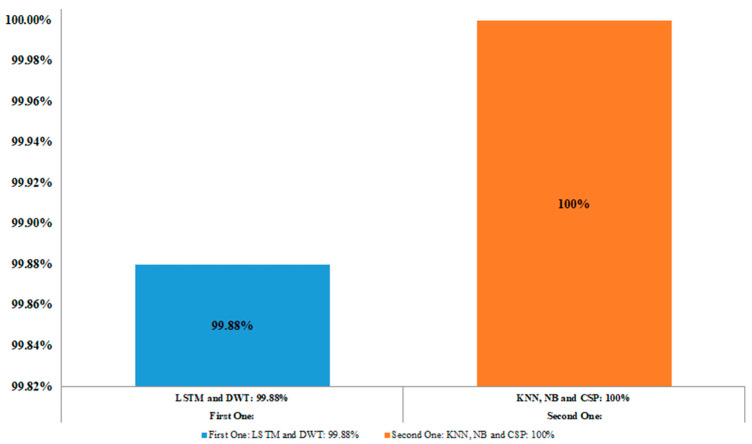
Average accuracy of data classification between the two best obtained results.

To create a comprehensive compilation in the research, the data obtained from the brain’s responses were analyzed using machine learning and statistical methodologies in all the literature and studies reviewed. Finding out how well the approach helps identify falsehoods was the primary goal of those trials. But lately, experts in this field have concentrated on integrating various technologies, methodologies, algorithms, and methods to improve the accuracy of EEG data categorization for lie detections. The outcome is beneficial and noteworthy, because compared to using just one approach, combining them may improve classification accuracy [2]. Additionally, the two most successful outcomes were achieved in the binary categorization of guilty and innocent groups utilizing ERP P300 in reaction to recognized and unrecognized faces via EEG. Using the LSTM and DWT techniques, the first one achieved an average data classification accuracy of 99.88% [16]. When applied to data that contained all channels, the second one achieved the greatest average data classification with a rate of 100% using KNN, NB and CSP techniques [27], as indicated in Figure 10. 

## 6. Discussion

Presently, to determine guilt or innocence, most scientists employ lie detection methods, including the CIT, DIT, GKT, EMD, TST, and Spatial Denoising Algorithm (SDA) [8,17,19,20,21,22,23,25,26,27,28,29]. These polygraphic approaches allow for the identification of psychophysiological activity, which is a field of psychology where only the guilty individual possesses knowledge of the specific details of the crime. These tactics utilize a sequence of inquiries to ascertain the subject’s identity and actions. However, several studies have been conducted for each of the described methodologies by simulating a criminal scenario to detect alterations in the cognitive components of EEG brain activity. The most often-employed approach for examining an individual’s deceptive conduct is the CIT technique, which relies on the ERP P300 paradigm. This is where the analysis of individual stimulus reactions takes place. If the P300 waveform is present, it can be inferred that the patient is engaging in deception. Unlike a polygraph, it is more difficult to fool, manipulate, or repress [17,19,20,21,22,23,25,26,27,29]. In the traditional CIT model, individuals are exposed to three types of stimuli: probes, targets, and irrelevant stimuli.

Nevertheless, most of the prior research and development has been on visual stimulus analysis through face recognition. In addition, there have been efforts to address topics such as autobiographical details, audiovisual stimuli, name recognition, interviews, and the identification of crime scene artefacts. Furthermore, several studies were conducted using simulated criminal situations, which involved elements such as the victim’s facial, a weapon used in the murder, the name of the accomplice, or a stolen item, among others. This is where we determine whether the individual was involved in the event or has knowledge of the crime scene or the specific object in question. Moreover, the P300 component is often assessed using the Fz, Cz, and Pz electrodes positioned along the central axis of the skull [1,74,75]. Furthermore, prior research utilizing EEG signals as its basis has shown that this component’s amplitude is the greatest and most effective in the frontal and prefrontal channels, including Fp1, Fp2, F3, F4, and Fz. Meanwhile, the moderate amplitude is in the parietal and central lobe channels, including P3, P4, Pz, and Cz. However, the baseline regions are in the occipital channels, including O1 and O2. Nevertheless, most researchers have narrowed their emphasis to studying a single Pz channel in the parietal region, the exact location at which the ERP P300 amplitude is greatest [1,73,75].

The evolution of wearable devices with EEG sensors has made this equipment more approachable and simpler to use. Over the past few years, researchers in this area have made use of a variety of devices, including the Emotiv Insight Pro, EasyCap, ElectroCap, Neuroscan, Biosemi, Wearable EMOTIV Headset, Mobile Brain Wear Headset, Brain Vision Recorder and Analyzer, each with a unique number of channels for collecting EEG data [8,16,17,18,19,20,21,22,23,24,25,26,27,28,29]. However, the evidence put forth in this review and numerous other studies indicate that recent advancements in the field of neuroscience empower scientists to discern data lodged in the brain might establish a noninvasive, truthful, and precise correlation between perpetrators and a particular offence. As a result, this approach can expedite, improve the precision, and streamline the resolution of cases while also affording blameless individuals a free-of-strain, dependable, and non-intrusive method of obtaining an exception.

Despite the utilization of contemporary techniques and approaches, complete accuracy in lie detection has nevertheless been attained at 100%. Even though considerable progress has been made in terms of the precision of classification through certain research endeavors, there remain numerous prospects for enhancement, including, but not limited to, real-time application, optimal classification accuracy, cost-effectiveness, enhanced availability, and reduced time consumption. It may be critical to employ techniques for element extraction, selection, and classification, given that distinct approaches are appropriate for various types of data processing. When choosing techniques for extracting and classification, factors such as the magnitude of the datasets, the nature of the stimulus, or the experimental protocol are taken into consideration. Choosing a classifier can be difficult because the computational difficulty and data processing duration of each algorithm differ.

### 6.1. Evaluating and Discussing Feature Extraction Algorithms for Lie Detection Studies

Since feature extraction from EEG signals is important for various applications such as BCIs, emotion recognition for lie detection, and cognitive state monitoring, the basic feature extraction algorithms for all studies conducted on EEG lie detection are evaluated and discussed along with their performance, effectiveness, purpose, use cases, and impact on the results. The DWT method is particularly effective for analyzing non-stationary signals as it efficiently captures both time and frequency features [16,17]. DWT decomposes signals into different frequency bands while preserving time information, making it suitable for signal denoising, feature extraction, biomedical signal processing, speech processing, and fault detection [18]. It enhances time-frequency localization and improves feature extraction for classification tasks [21,24]. In addition, the FFT is known for its high efficiency in computing frequency components, and it is effective for analyzing stationary signals [16,17]. It converts time-domain signals to the frequency domain and is commonly used for spectral analysis, vibration analysis, and audio/speech signal processing [19,20]. While it successfully identifies dominant frequency components, it lacks time localization, which may limit its application in certain scenarios [21,24]. However, SMs are simpler and computationally inexpensive, making them effective for estimating central tendency and providing insights into data distribution. They extract statistical features such as mean, variance, and standard deviation, which help compute the average of a dataset. SM is utilized for EEG analysis and fault diagnosis, aiding in classification and trend analysis [16].

Moreover, HSPs are quick and effective for EEG signal analysis. They measure signal mobility and complexity. They are used for EEG-based BCI and emotion detection. They differentiate between normal and abnormal signal patterns and different cognitive states [17,23]. Additionally, entropy measures the randomness or uncertainty in a system and quantifies signal complexity or information content. It is commonly used in EEG complexity analysis, cryptography, and image processing. Higher entropy values indicate greater complexity, while lower values suggest predictability. However, amplitude is a straightforward and direct measure of signal strength, which assesses either the peak or mean amplitude of a signal. It is utilized in vibration analysis and biomedical signal analysis, with high amplitude generally indicating stronger signal energy. While average amplitude is particularly effective for steady-state signals, it calculates the average of the absolute amplitude values. This measure is beneficial for signal characterization and biomedical analysis as it reduces the impact of noise compared to raw amplitude readings. In addition, P2P measures the full range of signal variation by calculating the difference between the maximum and minimum values in a signal. It is used in vibration monitoring and EEG signal analysis, providing insights into signal variability, which can be influenced by noise. Peak-to-peak time (P2PT) is valuable for time-domain characterization, as it measures the time interval between peak values. This measure finds application in biomedical signals and fault diagnosis, helping to identify periodicity and variations in signal behavior. However, approximate entropy assesses complexity in time series data by evaluating signal regularity and predictability. It is particularly useful in heart rate variability and EEG analysis, with lower values indicating more regular signals and higher values suggesting greater complexity [18].

On of the feature extraction method EMD is an adaptive and efficient method for decomposing complex and non-stationary signals into simpler oscillatory modes. It extracts IMFs from the signals and is commonly used in applications such as EEG, fault diagnosis, and seismic data analysis. EMD enhances time-frequency and trend analysis. However, it can suffer from mode mixing [19,26]. On the other hand, WT provides multi-resolution analysis and is suitable for both stationary and non-stationary signals. It analyzes signals across different frequency bands, time scales, and resolutions [19,25]. It is widely used in fields including image compression, biomedical signal processing, and speech processing, as it improves feature extraction with better time-frequency localization [19,29]. Additionally, STFT captures the time-frequency characteristics of signals but has a fixed resolution. It is preferable to the FFT for analyzing non-stationary signals, as it divides signals into short segments for Fourier analysis. STFT is commonly used in EEG analysis, speech processing, and audio classification, providing a trade-off between time and frequency resolution [8,20]. However, ICA extracts independent signal components, thus removing artefacts and noise from mixed signals. It effectively separates overlapping signals and is applied in EEG artefact removal, blind source separation, and financial data analysis. This method enhances signal quality and improves classification accuracy by isolating meaningful components [20].

GA, one of the optimization methods, is an effective method for feature selection and optimization, which uses an evolutionary approach to search for optimum solutions by selecting the best feature subsets. It is widely used in various applications, including feature selection, classification, image processing, biometric systems, and neural network optimization. While it effectively reduces dimensionality and improves performance and accuracy, GA does require significant computational resources. Additionally, SSA detects changes in the slope of a signal. This technique is particularly useful for analyzing transient signals, as it focuses on slope changes. SSA finds applications in seismic signal processing and significantly enhances event detection. ALAR measures changes in timing and intensity by detecting amplitude variations, which help identify amplitude-based features. It is commonly used in neurological studies, playing a crucial role in disorder diagnosis [21]. On the other hand, the PLV measures phase synchronization and captures brain connectivity. It evaluates the connectivity between signals and quantifies phase relationships. PLV is particularly valuable in EEG-based neuroscience studies and coherence analysis. A high PLV indicates strong phase coupling, thereby enhancing the analysis of connectivity in brain signals [22]. In addition, the ReLU is very fast and computationally efficient and prevents vanishing gradient issues but dead neuron issues. It is an activation function in neural networks and is used for DL models, speeds up training and improves learning efficiency [24]. At the same time, the GDA is another efficient optimization method that minimizes error in learning models. It is updated model weights iteratively and used for DL and neural networks. It improves model accuracy and convergence speed [25].

The hybrid combination combines multiple techniques for better performance and increased robustness and provides robust feature extraction to enhance classification accuracy. It is used for multi-modal analysis, biomedical, speech, and image processing. However, Burg’s method estimates power spectral density and is suitable for short data segments, and it has better resolution than FFT, which computes autoregressive model coefficients. It is used for EEG and seismic signal processing and provides accurate spectral estimates and breaks signals into different frequency bands. Moreover, IMFs extract individual oscillatory components and adaptive decomposition that are obtained from EMD. It is used for biomedical signal processing and enhances non-stationary signal analysis. Additionally, the HT extracts the analytic signal and is very useful in phase and envelope analysis, which computes instantaneous frequency and amplitude. It is used for EEG, and speech analysis, and enhances phase-based and frequency-based analysis [26]. The CSP filter is effective in optimizing variance for EEG classification and maximizes variance differences between classes and enhances discrimination in BCI. On the other hand, WPT improves frequency resolution and is more detailed than DWT, provides better frequency resolution, and further decomposes frequency bands. It is used for audio, speech, and biomedical signal processing. It also improves detailed frequency feature extraction [28]. Additionally, the Binary Bat algorithm is very good for optimization problems, inspired by bat echolocation, used in feature selection, and finds optimal feature subsets. It is used for classification tasks and EEG-based biometric authentication. It improves computational efficiency and accuracy. 

However, these feature extraction techniques and methods are used in different applications, such as biomedical signal analysis, speech processing, ML, and neural networks. Their choice depends on the specific requirements of the task, such as computational efficiency, accuracy, robustness, and interpretability [8].

### 6.2. Shortcomings of EEG-Based Lie Detection Studies

Although EEG-based lie detection studies have gained attention due to their potential to assess deception through brain activity, there are still several shortcomings associated with these studies. However, the most significant shortcomings of our conducted studies include the following [8,16,17,18,19,20,21,22,23,24,25,26,27,28,29]:

i. **Low Spatial Resolution:** EEG records brain activity at the scalp level and provides high temporal resolution but poor spatial resolution, and therefore cannot localize frontal lobe activity precisely, making it difficult to pinpoint the exact brain regions responsible for deception. Unlike hybrid EEGs, which could address spatial resolution issues, and fMRI, which can localize activity with greater precision, EEG signals are averaged across broad cortical areas, limiting the ability to distinguish specific neural correlates of lying [16,17,18,19,20,21,22,23].
ii. **Susceptibility to Artifacts:** EEG signals are highly sensitive to noise and artefacts from muscle movements, eye blinks, external electrical interference, and even minor head movements, which can distort the data and affect reliability [17,18,19,20,21,22,23,24].
iii. **Individual Differences:** EEG signals vary significantly between individuals due to differences in brain structure, cognitive strategies, and psychological states. What constitutes a deception-related brain response in one person may not be the same in another, limiting the generalizability of findings and making it challenging to develop a universal deception detection model [18,19,20,21,22,23,24,25].
iv. **Cognitive Complexity of Deception:** Lying is a complex cognitive and emotional process that involves multiple brain functions, including those influenced by memory, executive control, intention, personality traits, and emotional regulation. EEG may capture neural responses associated with these processes, but it cannot differentiate between deception and other cognitive activities like (anxiety, stress, or recall difficulty), leading to false positives [19,20,21,22,23,24,25,26].
v. **Inconsistency across Studies:** Many EEG-based lie detection studies show inconsistent results, partly due to differences in study design, task paradigms, and analysis methods. Variability in the stimuli used to induce deception and the classification methods can lead to contradictory findings, reducing the reliability of conclusions [20,21,22,23,24,25,26,27].
vi. **Difficulty in Establishing Reliable Biomarkers:** Identifying universal EEG-based lie detection remains a challenge. While certain waveforms like (P300 and N400) have been linked to deception, these markers are also associated with other cognitive processes, making it difficult to establish a definitive neural indicator of lying [21,22,23,24,25,26,27,29].
vii. **Limited Ecological Validity:** Most EEG-based deception studies are conducted in controlled lab environments with artificial tasks, which may not reflect real-world deception. Differences in motivation, emotional states, and stakes influence brain activity. The stakes are often low in experimental settings, meaning the neural mechanisms involved in deception may differ from those in high-stakes, real-life situations [22,23,24,25,26,27,28,29].
viii. **Ethical and Legal Concerns:** Using EEG for lie detection raises ethical concerns, including privacy violations and the risk of misclassifying innocent individuals. The legal admissibility of EEG-based lie detection is also questionable, as the reliability of brainwave-based deception detection is not yet robust enough for forensic or legal contexts, applications and inadmissibility in courts [8,23,24,25,26,27,28,29].

While EEG offers a promising tool for studying deception, its limitations, such as low spatial resolution, susceptibility to artifacts, ethical concerns, lack of real-world applicability, and other limitations, highlight why EEG-based lie detection remains an experimental tool rather than a reliable forensic method, which is why this study needs further research and methodological improvements. Additionally, future studies should address these shortcomings to enhance the reliability and validity of EEG-based lie detection.

### 6.3. Limitation of High-Accuracy Claims

The claims of high accuracy derived from limited datasets might be deceptive owing to several constraints that compromise the reliability and generalizability of the findings. This encompasses overfitting, wherein models trained on small datasets may memorize patterns unique to the training data instead of acquiring generalizable patterns, resulting in artificially inflated accuracy on the training set that fails to extend to new data, leading to subpar performance on unseen data and a misleading perception of model effectiveness. Secondly, insufficient statistical power and representation, along with small datasets, diminish confidence in model evaluation and elevate the risk of Type I and Type II errors in reported accuracy as a genuine reflection of performance. This results in limited variability and sample diversity, causing high variance in model performance across different splits and complicating the differentiation between genuine patterns and noise. Thirdly, inadequate generalization, elevated accuracy on a limited sample does not ensure that the model would excel on bigger, real-world datasets. This is because tiny datasets may fail to encapsulate the complete intricacy of the issue domain, perhaps omitting significant edge cases and infrequent occurrences. Fourthly, evaluation bias in small datasets can significantly affect evaluation metrics, as a limited number of correctly or incorrectly classified instances drastically alter accuracy, leading to potentially misleading conclusions based on random chance rather than actual model performance. Fifth, reproducibility challenges arise since the limited dataset frequently leads to inconsistent outcomes between iterations due to sensitivity to beginning circumstances or train-test divisions, complicating the replication of results, which is essential for scientific rigor. Ultimately, confirmation bias and cherry-picking may lead researchers to choose models or setups that perform optimally on the limited sample. Moreover, inadvertently emphasizes advantageous results without enough verification, particularly if the dataset is improperly segmented or randomized [83,84].

## 7. Conclusions

The focus of this research was examining current scientific studies on utilizing EEG to identify lie by analyzing the ERP P300 paradigm’s reaction to visual stimuli of recognized and unrecognized faces. The CIT approach was the predominant technique utilized for assessing an individual’s deceptive conduct. However, the scientists mostly used a variety of approaches, including the FFT and DWT methods, for feature extractions in this particular setting. Furthermore, the SVM, LDA, MLFFNN and KNN methods were often utilized as classifiers. Another significant discovery is that the researchers in these fields have lately concentrated on merging several approaches for EEG-based lie detection to improve average data classification accuracy. Numerous new developments from a wide range of fields have been made possible by the rapid advancements in EEG mobile devices in recent years. This research study provides a comprehensive analysis of the latest techniques utilized in the field to develop a highly effective fraud detection system that uses visual facial cues.

## Figures and Tables

**Table 1:** Examination and comparison of existing approaches and methods.

Used Device and Protocol	Dataset (no. Participants)	Preprocessing Approaches	Feature Extraction Approaches	Classification Methods	Accuracy Scores	Referece
Emotive Insight Pro	LieWaves (27)	ATAR and OSW	DWT + FFT + SM	CNN, LSTM, CNN-LSTM	99.88%	[16]
Polygraph and Brain Vision Recorder and Analyzer Devices with GKT and CIT	Own data (10)	BPF	DWT, FFT, and Hjorth’s Statistical Parameters (HSP)	LDA, SVM, MLFFNN, KNN, and NB	92.40%	[17]
Wearable EMOTIV Headeset and Brain Vision Recorder and Analyzer Devices	Knowledge (30)	Principal Component Analysis (PCA), and Smart Signal Processing Techniques (SSPT)	DWT, entropy, amplitude, average amplitude, peak-to-peak, peak-to-peak time, and approximate entropy	SVM	83.00%	[18]
Polygraph and Brain Vision Recorder and Analyzer Devices with CIT	Own data (10)	BPF Device, Binary Bat, and Conventional Bat	FFT, EMD, and WT	LDA, SVM, KNN, NB, and MLFNN	96.80%	[19]
ElectroCap Device with CIT	Own data (20)	BPF Device	FFT, STFT, and Independent Component Analysis (ICA)	LC, K-means Clustering, XGBoost, SVM, and ANN	86.00%	[20]
Polygraph and Brain Vision Recorder and Analyzer Devices with GKT and CIT	Own data (49)	BPF Device, and Visual Inspection of EOG data	DWT, FFT, GA, Slope Signal Alteration (SSA), and Absolute Latency/Amplitude Ratio (ALAR)	LDA	91.83%	[21]
Neuroscan Synamps Amplifier Recording Device with CIT	Own data (20)	BPF Device, EEG LAB, and PS	PLV	SVM	88.05%	[22]
EasyCap Device with CIT	Own data (10)	BPF Device	HSP	KNN	96.70%	[23]
Mobile Brain Wear Headset Device and Emotive Insight Pro	Dryad and Lie Detection datasets (30)	BPF Device	Rectified Linear Unit (ReLU) Activation Function, DWT, and FFT	CNN	84.44% for Dryad, and 82.00% for Lie detection datasets	[24]
EasyCap Device with CIT	Own data (10)	BPF Device	WT, and Gradient Descent Algorithm (GDA)	MLFFNN	83.10%	[25]
EasyCap Device with CIT	Own data (33)	BPF Device	EMD + Burg’s Wavelet Decomposition (BWD) + Intrinsic Mode Functions (IMF) + Hilbert Transform (HT)	SVM	99.44%	[26]
Polygraph and Brain Vision Recorder and Analyzer Devices with CIT	Own data (10)	BPF Device	CSP	Fuzzy, LDA, MLFFNN, KNN, SVM, and NB	LDA = 96.67%, MLFFNN and SVM = 98.33, and NB, and KNN = 100%	[27]
EasyCap Device with CIT	Own data (10)	BPF Device	WT	DBN	81.03%	[29]
EasyCap and Brain Vision Recorder and Analyzer Devices with CIT	Own data (20)	BPF Device	WPT	LDA	91.67%	[28]
EasyCap and Brain Vision Recorder and Analyzer Devices with CIT	Own data (10)	BPF Device	STFT, and Binary Bat	ELM	88.30%	[8]

**Table 2:** Comparison and overview of several common algorithms.

Algorithm	Avg. Time Complexity	Worst-Case Complexity	Noise Robustness	Real-Time Applicability
QuickSort	O(nlogn)	O(n^2^)	Low–Medium	Medium
MergeSort	O(nlogn)	O(nlogn)	Low	Medium
Kalman Filter	O(n)	O(n)	High	High
Extended Kalman Filter	O(n^2^)	O(n^2^)	High	High
SVM (linear)	O(n)	O(n^2^)	Medium–High	Medium
SVM (non-linear kernel)	O(n^2^. d)	Up to O(n^3^)	Medium	Low
CNN (Inference)	Varies (e.g., O(n^2^))	Hardware dependent	High	High (if optimized)
LDA	O(nd^2^)	O(nd^2^)	Medium	High
PCA	O(nd^2^ + d^3^)	O(nd^2^ + d^3^)	Low–Medium	High
KNN	O(n. d) for query	O(n. d) for query	Medium–High	Low–Medium
K-Means	O(nkdi)	O(n^2^)	Low–Medium	Low–Medium
Naive Bayes	O(nd)	O(nd)	Medium–High	High
A* (A-star) Search	O(b^d^)	O(b^d^)	Medium	Medium–High
Random Forest (RF)	O(nlogn), t = trees	O(nlogn), t = trees	High	Medium–High
Logistic Regression (LR)	O(nd)	O(nd)	Medium	High

* means star.

## Data Availability

No new data were created in this study. Data sharing is not applicable to this article.

## List of Abbreviations and Symbols

ALAR	Absolute Latency/Amplitude Ratio
ADC	Analog to Digital Conversion
ACC	Anterior Cingulate Cortex
ANN	Artificial Neural Network
ADHD	Attention Deficit and Hyperactivity Disorder
ATAR	Automated and Adjustable Artefact Removal
BPF	Bandpass Filter
BAD	Bootstrapped Amplitude Difference
BCD	Bootstrapped Correlation Difference
BWD	Burg’s Wavelet Decomposition
CAR	Common Average Reference
CSP	Common Spatial Pattern
CIT	Concealed Information Test
CF	Connectivity Features
CQT	Control Question Test
CNN	Convolutional Neural Networks
DIT	Deceit Identification Test
DBN	Deep Belief Network
DL	Deep Learning
DWT	Discrete Wavelet Transform
DCM	Dynamic Causal Modeling
EEG	Electroencephalography
EOG	Electrooculography
ER	Emotion Recognition
EMD	Empirical Mode Decomposition
ERP	Event-Related Potential
EPs	Evoked Potentials
ELM	Extreme Learning Machine
FFT	Fast Fourier Transform
FD	Fractal Dimension
fMRI	functional Magnetic Resonance Imaging
GA	Genetic Algorithm
GDA	Gradient Descent Algorithm
GKT	Guilty Knowledge Test
HT	Hilbert Transform
HSP	Hjorth’s Statistical Parameters
ICA	Independent Component Analysis
IMFs	Intrinsic Mode Functions
KNN	K-Nearest Neighbor
LC	Linear Classifier
LDA	Linear Discriminant Analysis
LR	Logistic Regression
LSTM	Long/Short Term Memory
ML	Machine Learning
MRI	Magnetic Resonance Imaging
mPFC	medial Prefrontal Cortex
MI	Motor Imagery
MLFFNN	Multilayer Feed Forward Neural Network
NB	Naive Bayes
OSW	Overlapping Sliding Window
P2P	Peak-to-Peak
P2PT	Peak-to-Peak Time
PLV	Phase Locking Value
PS	Phase Synchronization
PSD	Power Spectral Density
PRISMA	Preferred Reporting Items for Systematic Reviews and Meta-Analyses
PFC	Prefrontal Cortex
PCA	Principal Component Analysis
RF	Random Forest
ReLU	Rectified Linear Unit
RNN	Recurrent Neural Network
STFT	Short-Time Fourier Transform
SNR	Signal-to-Noise Ratio
SSA	Slope Signal Alteration
SSPT	Smart Signal Processing Techniques
SDA	Spatial Denoising Algorithm
SDF	Spatial Domain Feature
SM	Statistical Method
SSVEPs	Steady State Visually Evoked Potentials
SVM	Support Vector Machine
TST	Three-Stimulus Technique
WPT	Wavelet Packet Transform
WT	Wavelet Transform
WV	Weighted Voting
